# Identification of the Infrapatellar Branch of the Saphenous Nerve for Treatment Using a Peripheral Nerve Stimulator: A Technical Report

**DOI:** 10.7759/cureus.4668

**Published:** 2019-05-15

**Authors:** Brian McLean

**Affiliations:** 1 Pain Department and Interdisciplinary Pain Management Center, Tripler Army Medical Center, Kaneohe, USA

**Keywords:** cryoablation, pain management, anterior knee pain, interventional pain management, peripheral nerve stimulation, pain, nerve block

## Abstract

The infrapatellar branch of the saphenous is becoming a common therapeutic target for the diagnosis and treatment of anterior knee pain. It is a nerve commonly injured during knee surgeries, resulting in neuroma formation and chronic neuropathic pain states, and can also transmit nociceptive input in patients with non-surgical anterior knee pain of multiple etiologies. After diagnosing infrapatellar saphenous neuralgia, the nerve is safely ablated using radiofrequency ablation, neurolytic solutions, and, most recently, cryoablation using the handheld iovera® cryoablation system (Myoscience, Inc. Fremont, CA). The iovera® technology benefits from procedural simplicity in that the nerve doesn’t specifically need to be identified and the described technique involves treating a long line over which the infrapatellar branch of the saphenous nerve is expected to course. However, there is significant variability in the course of the nerve and much of the area treated misses the actual location of the nerve, wasting time and potentially increasing patient discomfort and risk of complications. To address these limitations we endeavored to identify a way to more precisely treat the specific location of the nerve thereby optimizing treatment success and procedural simplicity. Using a MiniStim® peripheral nerve stimulator (Halyard Health, Inc., Georgia, US) to scan for the nerve along the previously described treatment line, we have been able to identify a more precise location of the nerve and optimize the treatment target area. This non-invasive identification technique has, to our knowledge, not been previously described.

## Introduction

Chronic non-surgical anterior knee pain is a common condition and a major source of disability, with limited long-term treatment options after a patient has failed physical therapy [1-2]. Many techniques have been suggested and trialed to include oral medications, bracing, acupuncture, transcutaneous electrical nerve stimulation (TENS) therapy, massage, corticosteroid injections, viscous supplementation, prolotherapy, platelet-rich plasma therapy, and nerve ablation procedures [3-5]. Each intervention has significant limitations and variable outcomes with regard to patient comfort, duration of action, and chance of treatment success. In the patients that we manage with chronic non-surgical anterior knee pain who have failed physical therapy and the other conservative modalities of pain management listed above, we next consider palliative neuroablative techniques. Initially, we employed radiofrequency ablation to the geniculate nerves and peripheral nerves of the knee, however, due to observed complications, such as loss of proprioception and post-procedural neuritis, we have moved first to trial diagnostic infrapatellar branch of the saphenous (IPBS) nerve block followed by cryoablation of this branch with the iovera® device (Myoscience, Inc. Fremont, CA). This procedure has been demonstrated to be effective in patients with symptoms of knee arthritis [4], and we also find it effective for patients with chronic non-specific anterior knee pain.

## Technical report

**Figure 1:**
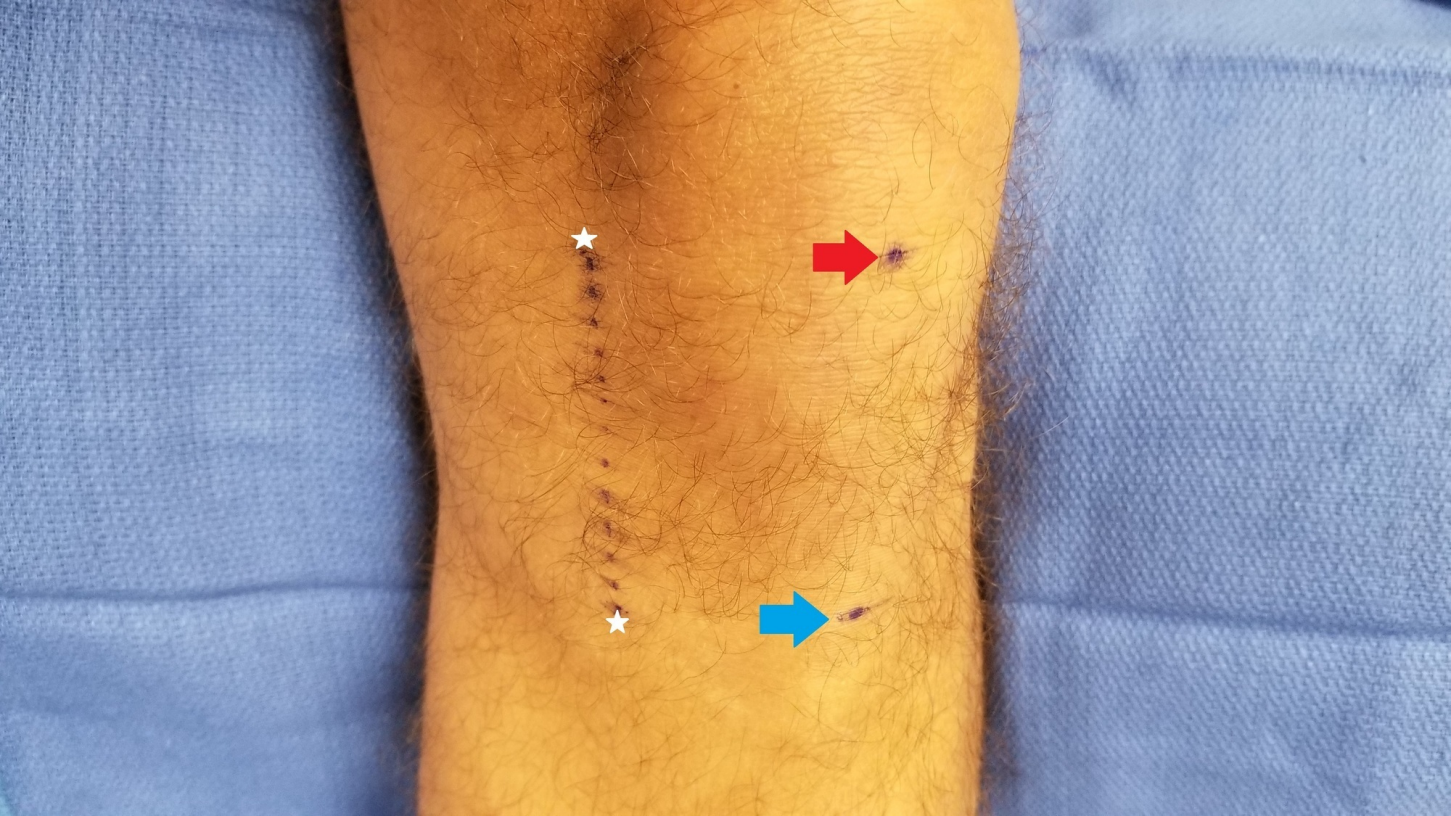
Typical iovera® treatment location Red Arrow: Inferior pole of the patella; Blue Arrow: Tibial tubercle; White Stars: iovera® procedure recommended treatment line

In the procedural instructions for the iovera® device and in studies evaluating effectiveness, the recommended treatment location is a line drawn 5 cm medial from the inferior pole of the patella and tibial tubercle, as seen in Figure 1. The purpose of this recommendation is to simplify the procedure and encompass the variable course of the infrapatellar branch of the saphenous nerve. Ackmann et al. [6] performed a cadaver study to provide an anatomic analysis and demonstrate the risk of nerve injury during knee surgery. Their analysis found four anatomical variations along a line between 15 and 40 mm medial to the inferior pole of the patella with a fairly even distribution as to the variation in the location of the infrapatellar branch. Kim and Tran et al. [7-8] also conducted cadaver studies to determine the optimal stimulation and recording site for the IPBS nerve and similarly found that there was variability in the location of the branch. Depending on the height of the patient, this line can span many centimeters, and in order to treat the entire line, as seen in Figure 1, eight or more cycles of cryoablation need to be performed. While multiple treatment sites may catch more terminal branches, our goal is to treat the infrapatellar branch of the saphenous specifically prior to branching and deliver all cryoablation at the target nerve to improve procedural outcome and avoid freezing of extraneous tissue.

**Figure 2:**
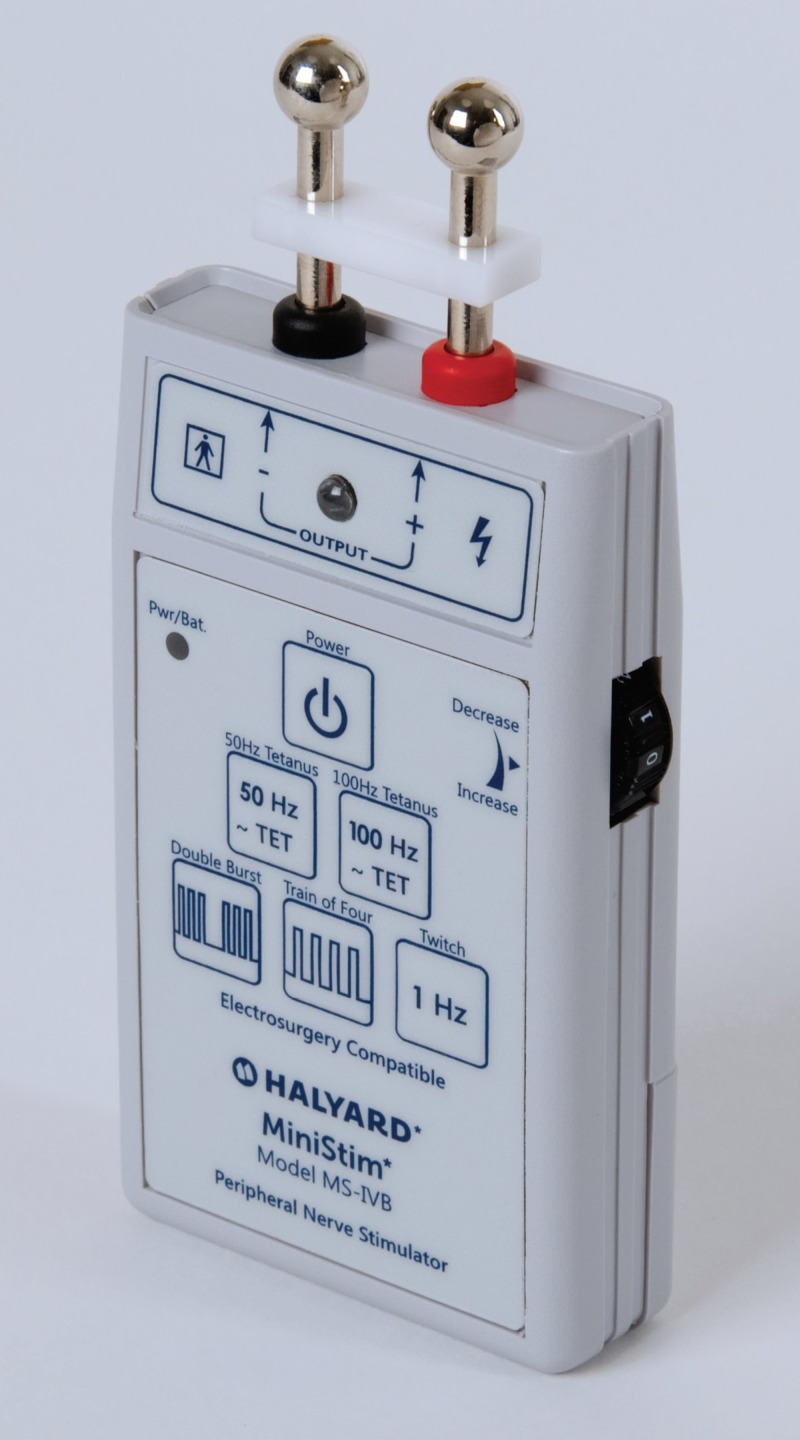
Halyard Ministim® MS-IVB model Halyard Ministim® MS-IVB model: Halyard Health, Inc., Georgia, US

The MiniStim® Model MS-IVB (Halyard Health, Inc., Georgia, US), as seen in Figure 2, is a commonly used and relatively inexpensive peripheral nerve stimulator used by anesthetists to monitor the effects of skeletal muscle relaxants during general anesthesia by non-invasively stimulating peripheral nerves below the skin surface. When typically used for monitoring neuromuscular blockade, a motor stimulation is used at 2 Hz to cause the contraction of the muscle innervated by the targeted nerve. The device also has a surface probe attachment, which allows scanning over the skin and has a 50 Hz tetanus setting as well as an intensity control knob. This allows for stimulation at an acceptable threshold for the awake patient to sense nerve stimulation as a comfortable buzzing sensation while avoiding unnecessary discomfort or invasive needles for stimulation. Another feature of this device helpful in determining the location of the IPBS is the output stimulus pulse/open lead Indicator. This indicator will remain off while scanning for the nerve as it cannot detect current between the positive and negative terminals due to the relatively high impedance of the skin and adipose tissue. However, when the probe is over a nerve, the tissue resistance will drop and the indicator will light up as a further confirmation that current is flowing between the terminals. Note that in some patients who are more sensitive to the stimulation, they may feel the stimulation and give you verbal feedback before the device will light up.

**Figure 3:**
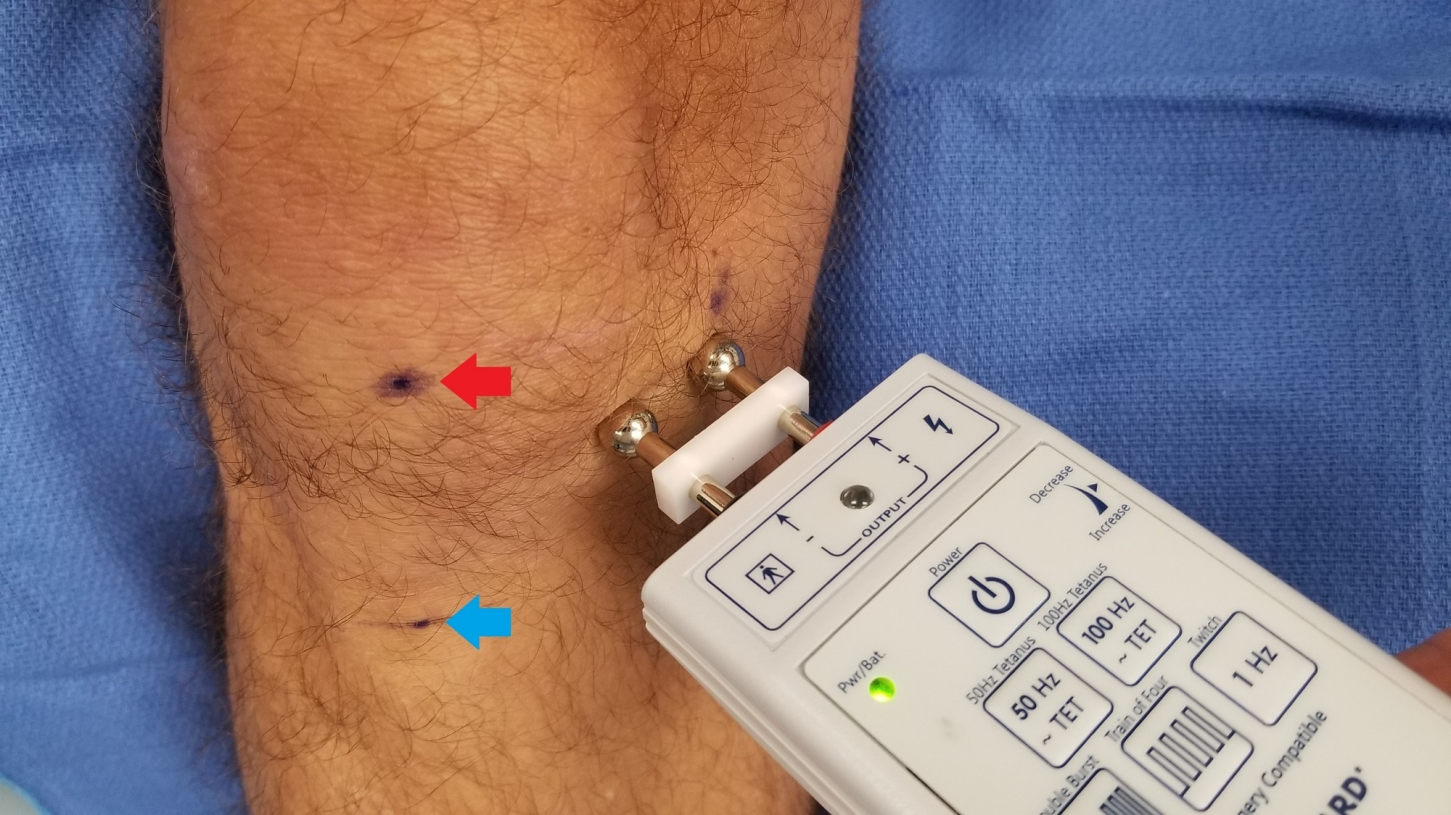
Recommended simulator placement Red Arrow: Inferior pole of the patella; Blue Arrow: Tibial tubercle

**Figure 4:**
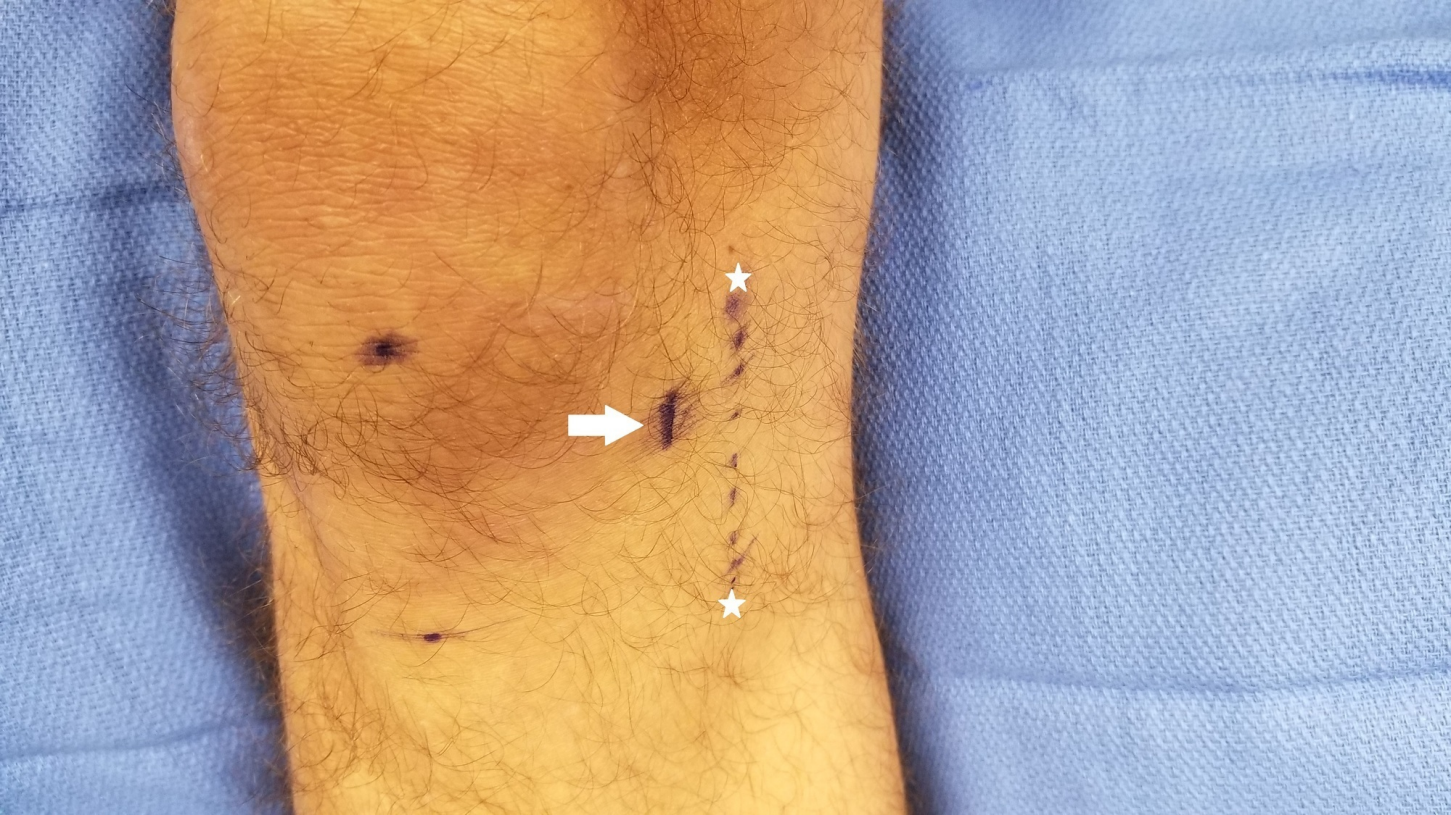
Comparison of original and adjusted treatment lines White Stars: Original iovera® recommended treatment line; White Arrow: Peripheral nerve stimulator identified treatment line

To employ the Ministim® to identify the IPBS nerve location, we recommend the following procedure. Identify the inferior pole of the patella and place the bipolar surface probe with the negative terminal approximately 5 cm medial to this location and the positive probe more medial, as seen in Figure 3. Start with the stimulus amplitude dial set at approximately two and hold down the 50 Hz tetanus button, instructing the patient to tell you when they feel a pinch or tingling sensation. Scan up and down a vertical line approximately 5 cm from the patella and tibial tuberosity. If the patient does not feel stimulation at this setting, gradually increase the output in approximately 0.5 increments until the patient detects a sensation at one of the terminals. We have found that for our patient population, the stimulation threshold has been at approximately three to four on the dial. After perception of the stimulus, make a slow and deliberate scan of the area medial to the patella and instruct the patient to tell you when they feel stimulation travel to the center of their knee or pain location as opposed to local stimulation. The patient should sense the stimulation traveling to the center of the knee more than directly under the area of stimulation. Where this happens, mark the location with a skin marker. Next, confirm the location by stimulating 0.5 cm above and below this mark to confirm the precise location of the point of maximal stimulation. After marking this location, we use a 30-gauge 0.5-inch needle to then anesthetize this point with 1 ml of 2% lidocaine or 0.5% ropivacaine for the diagnostic block and then provide them with a pain diary and instructions to perform activities that would normally exacerbate their knee pain. If they have at least 75% improvement, we will offer them a trial of cryoablation. For the formal cryoablation procedure, the same exact localization technique is used as above and then 1 ml of 2% lidocaine is utilized over the skin marking. Cryoablation is then performed using the iovera® smart tip, treating for two to four cycles with overlapping placements. An image of the typical treatment area is shown in Figure 4.

## Discussion

Quality outcomes for neuroablative pain palliation procedures require precise identification of the target nerve location. This non-invasive technique to identify the infrapatellar branch of the saphenous nerve is well-tolerated and expedient. Identification of the target nerve location significantly decreases the necessary treatment area. In addition to saving time, we hypothesize that patients treated using this method should have higher treatment success with a longer duration of pain relief. Anecdotally, we have used this treatment technique and observed immediate response and pain relief after the cryoablation procedure, presumably indicating accurate treatment of the infrapatellar branch of the saphenous nerve.

## Conclusions

Using this technique has decreased the overall treatment area as well as the procedural time, and we hypothesize that outcomes will be better given more targeted and overlapping treatments that should more robustly ablate the nerve. To confirm this hypothesis, a randomized trial comparing the two methods should be performed.
